# TRIM28 in cancer and cancer therapy

**DOI:** 10.3389/fgene.2024.1431564

**Published:** 2024-07-19

**Authors:** Kailang Li, Haifeng Wang, Bitao Jiang, Xiaofeng Jin

**Affiliations:** ^1^ Department of Oncology and Hematology, Beilun District People’s Hospital, Ningbo, China; ^2^ Department of Biochemistry and Molecular Biology, Zhejiang Key Laboratory of Pathphysiology, Medical School of Ningbo University, Ningbo, China

**Keywords:** TRIM28, ubiquitination, SUMOylation, transcription inhibitor, tumorigenesis, cancer therapy

## Abstract

TRIM28 (tripartite motif protein 28) was initially believed to be a transcription inhibitor that plays an important role in DNA damage repair (DDR) and in maintaining cancer cellular stemness. As research has continued to deepen, several studies have found that TRIM28 not only has ubiquitin E3 ligase activity to promote degradation of substrates, but also can promote SUMOylation of substrates. Although TRIM28 is highly expressed in various cancer tissues and has oncogenic effects, there are still a few studies indicating that TRIM28 has certain anticancer effects. Additionally, TRIM28 is subject to complex upstream regulation. In this review, we have elaborated on the structure and regulation of TRIM28. At the same time, highlighting the functional role of TRIM28 in tumor development and emphasizing its impact on cancer treatment provides a new direction for future clinical antitumor treatment.

## 1 Introduction

PTMs (Post-translational modifications) play a crucial role in controlling the activity and stability of proteins in response to intracellular and extracellular stimuli (Venne et al., 2014). These modifications involve the cleavage of precursor proteins or covalent addition of modified groups (Venne et al., 2014; Ramazi and Zahiri, 2021). Ubiquitination is a prominent PTM that transfers ubiquitin to substrates through multiple enzymatic reactions, thereby promoting protein degradation and maintaining intracellular protein homeostasis (Sheng et al., 2021). Mechanistically, ubiquitin is activated by E1, coupled with E2, and then transferred to substrates with the help of E3 ligase (Sheng et al., 2021). Substrates labeled with ubiquitin are typically degraded by proteasomes (Sheng et al., 2021). Ubiquitin E3 ligase plays a crucial role in recognizing substrates and maintaining the ubiquitin processes. Dysfunction of ubiquitin E3 ligase can lead to abnormal accumulation or excessive degradation of substrates, which is closely related to the onset and progression of cancer (Sheng et al., 2021). SUMOylation is another important PTM, which is a small ubiquitin-like modification (Zhang and Zeng, 2020; Sheng et al., 2021). Similar to ubiquitination, SUMOylation requires E1 activation, E2 coupling, and E3 ligation to attach SUMO molecules to substrates (Sheng et al., 2021). Although SUMO E3 ligase is not necessary for SUMOylation, the dysregulation of SUMOylation E3 ligases also greatly affects the SUMOylation of substrates, promoting the occurrence of cancer (Li et al., 2023).

TRIM28, also known as KAP1 (Krüppel-Associated Box (KRAB)-Associated Protein 1) and transcription intermediary factor-β (TIF1β), is a RING (really interesting new gene)-type ubiquitin E3 ligase (Czerwińska et al., 2017a). Notably, TRIM28 catalyzes the poly-ubiquitinylation and degradation of various substrates, such as AMPK (AMP-activated protein kinase) AMPK) and RING finger LIM-domain-interacting protein (RLIM), also known as RNF12), by cooperating with the melanoma-associated antigen-encoding gene (MAGE), which plays an important role in the occurrence and development of tumors (Doyle et al., 2010; Pineda and Potts, 2015; Jin et al., 2021). Moreover, under certain conditions, TRIM28 also promotes SUMOylation of target proteins such as PD-L1 (programmed cell death ligand 1) (Ma et al., 2023). In addition, TRIM28 can serve as a skeletal protein that is recruited to chromosomes by Krüppel-related cassette zinc finger protein (KRAB-ZFP) and exerts a certain transcriptional inhibitory effect by binding to various chromatin remodeling factors (Sun et al., 2019). Emerging evidence suggests that inhibiting TRIM28 may enhance the efficacy of antitumor treatments (Tan et al., 2022; Han et al., 2023; Ma et al., 2023). In this review, we have elaborated on the structure and regulation of TRIM28. At the same time, highlighting the critical role of TRIM28 in cancer development and treatment provides new directions for future clinical antitumor therapies.

## 2 Structure feature and regulation of TRIM28

**FIGURE 1:**
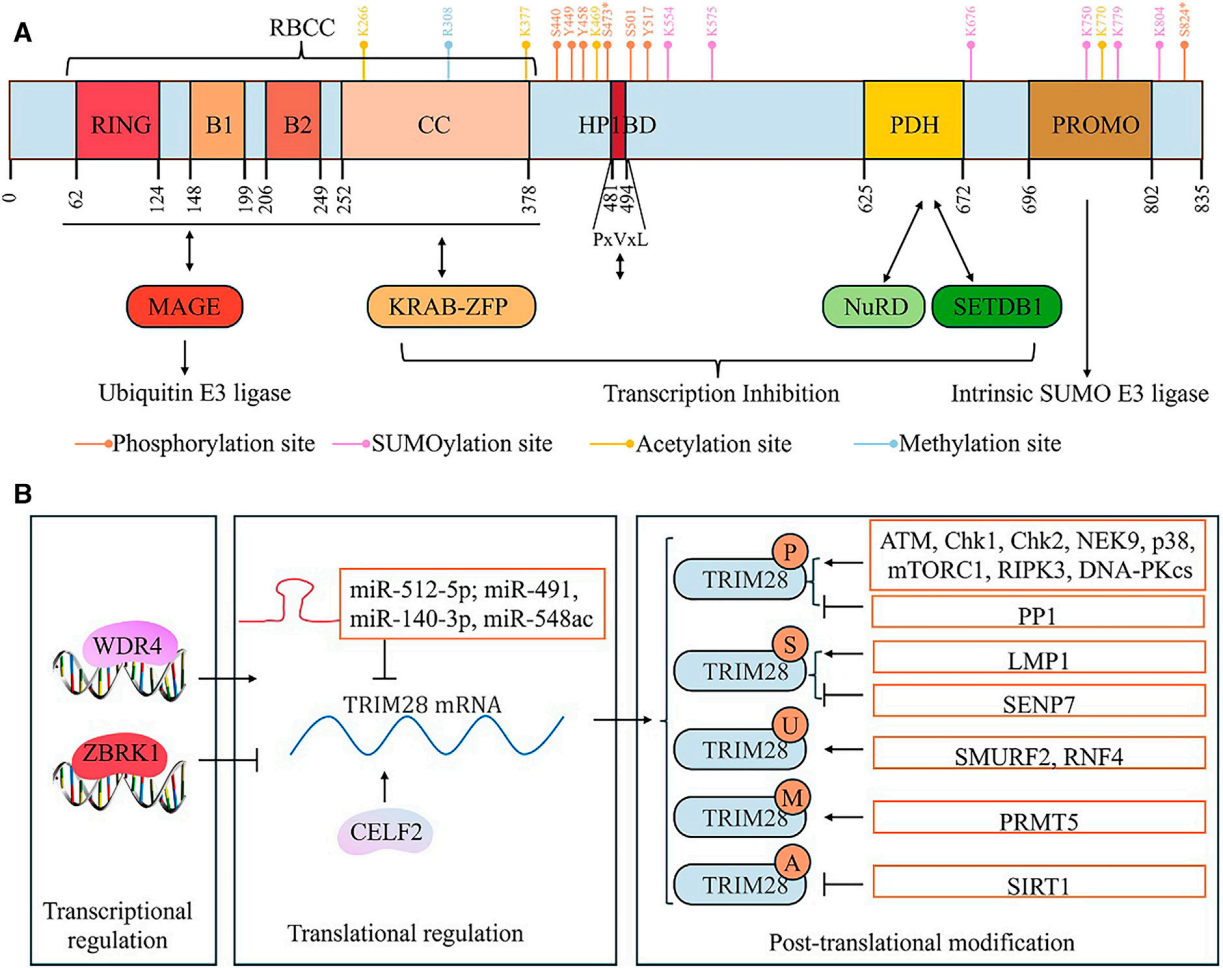
The structure and regulation of TRIM28. **(A)** The structure of TRIM28: TRIM28 consists of an N-terminal RING domain, two B-boxes (B1 and B2), a leucine zipper coiled-coil region (CC), an intermediate PxVxL pentapeptide domain, and a C-terminal plant homeodomain (PHD) and bromodomain. The RBCC domain, composed of the RING domain, B1 and B2 boxes, and CC domain, not only binds to the MAGE protein and functions as a ubiquitinated E3 ligase but also binds to the KRAB domain of the KRAB-ZNP protein. The PHD domain recruits NuRD and SETDB1 and the central PxVxL pentapeptide region binds to HP1. The transcriptional inhibitory activity of TRIM28 is closely related to its binding to SETDB1, NuRD, and HP1 proteins, but it is also influenced by SUMOylation modification of TRIM28 itself. The PHD domain has intrinsic E3 SUMO ligase activity. **(B)** The regulation of TRIM28.

The human *TRIM28* gene is located on chromosome 19 and contains 6,652 bases (International Human Genome Sequencing Consortium, 2004). TRIM28 is a nuclear protein with 835 amino acids that is composed of multiple domains (Figure 1A) (Peng et al., 2000). TRIM28 belongs to the TRIM family of proteins (Hatakeyama, 2017). Like other TRIM family proteins, the N-terminus of TRIM28 has a TRIM domain (also known as the RBCC domain) composed of an RNG domain, B-boxes (B1 and B2), and leucine zipper curled helical region (CC) (Peng et al., 2000; Hatakeyama, 2017). The B-box domain may be an important structure for the formation of TRIM28 polymers (Sun et al., 2019). In addition, the interaction between the RBCC region and the KRAB-ZFP transcription factor exerts the transcriptional inhibitory function of TRIM28 (Peng et al., 2007). Moreover, the RBCC region can bind to MAGE, thereby exerting the ubiquitination activity of TRIM28 (Xiao et al., 2014). Meanwhile, the MAGE-TRIM28 complex can ubiquitinate and degrade KRAB-ZFP family proteins, thus promoting downstream target gene transcription. However, since both MAGE and KRAB-ZFP bind to the RBCC domain of TRIM28 protein, KRAB-ZFP may competitively bind to TRIM28 protein, thereby inhibiting the ubiquitination activity of TRIM28 and subsequently inhibiting downstream gene transcription (Xiao et al., 2014).

The C-termini of TRIM family proteins have different structural domains, which is the basis for their protein specificity and classification of TRIM subfamilies (Hatakeyama, 2017). In the TRIM28 protein, the domains located at its C-terminus are the plant homeodomain (PHD) and bromodomain (Peng et al., 2000). Furthermore, the core of TRIM28 consists of a PxVxL pentapeptide domain (Peng et al., 2000). The PHD domain is a region rich in cysteine/histidine, which can recruit Nucleosome Remodeling Deacetylase (NuRD) and SET domain forked histone lysine methyltransferase 1 (SETDB1), thereby exerting chromosomal cohesion. The central PxVxL region binds to HP1 (Heterochromatin protein 1) (Sripathy et al., 2006). Currently, it is believed that PHD, bromodomain, and PxVxL regions synergistically form concentrated heterochromatin, leading to transcriptional inhibition (Sripathy et al., 2006; Xiao et al., 2014).

TRIM28 is regulated at several levels, including gene transcription, post-transcriptional translation, and PTM (Table 1) (Figure 1B). For example, WDR4 (WD repeat domain 4) can induce transcription of the *TRIM28* gene (Han et al., 2023). ZBRK1/ZNF350 (zinc finger and BRCA1-interacting protein with KRAB domain-1, also known as zinc finger protein 350) represses *TRIM28* transcription by suppressing its promoter activity (Lin et al., 2013). MicroRNAs (miRNAs) inhibit the expression of TRIM28 by binding to TRIM28 mRNA. miR-512-5p, miR-491, miR-140-3p, and miR-548ac have been found to reduce the levels of endogenous TRIM28 in gastric cancer (GC), glioblastoma, breast cancer (BC), and acute myeloid leukemia (AML) cells (Qi Z. et al., 2016; Zhou et al., 2019; Zou et al., 2021; Zhao et al., 2022). The RNA-binding protein CELF2 binds to TRIM28 mRNA, thereby promoting TRIM28 protein expression (Turchi et al., 2023).

**TABLE 1:** The regulation of TRIM28.

Regulation	Regulator	Regulate mode	Reference
Transcriptional regulation	WDR4	WDR4 acts directly on TRIM28 gene to promote the transcription of TRIM28 gene	Han et al. (2023)
	ZBRK1	ZBRK1 represses TRIM28 gene transcription through suppressing its promoter activity	Lin et al. (2013)
Translational regulation	miR-512-5p; miR-491, miR-140-3p, miR-548ac	MicroRNAs inhibit the translation of TRIM28 mRNA by target 3′UTR.	Qi et al. (2016a), Zhou et al. (2019), Zou et al. (2021), Zhao et al. (2022)
	CELF2	CELF2 efficiently binds to TRIM28 mRNA and promotes the expression of TRIM28 protein	Turchi et al. (2023)
Post-translational modification	DNA-PK	DNA-PK phosphorylates TRIM28 at the S824 site in a HIF dependent manner, thereby promoting the transcription of HIF target genes	Yang et al. (2022)
	p38MAPK	p38MAPK phosphorylates TRIM28 at S473, thereby contributing to cancer cell survival under conditions of sustained metabolic stress	Cheng et al. (2016)
	RIPK3	RIPK3 phosphorylates TRIM28 at S473, thereby promoting trans activation of NF-κB and other transcription factors	Park et al. (2021)
	mTORC1	mTORC1 phosphorylates TRIM28, thereby activating hTERT transcription	Agarwal et al. (2021)
	ATM	ATM phosphorylates TRIM28 at two residues (S473 and S824), thereby inhibiting the transcriptional inhibitory effect of TRIM28 and promoting DDR	Ziv et al. (2006)
	Chk1	Chk1 phosphorylates TRIM28 at S473 in response to ultraviolet radiation	Blasius et al. (2011)
	Chk2	Chk2 phosphorylates TRIM28 at S473 under etoposide or IR induced DNA damage	Magni et al. (2015)
	NEK9	NEK9 phosphorylates TRIM28 at the S824 and S473 sites, thereby enabling GC cells to obtain excessive migration ability	Lu et al. (2023)
	PP2A	PP2A de- phosphorylates TRIM28 at S824 site	Mita et al. (2016)
	SIRT1	SIRT1 promotes the deacetylation of TRIM28, thereby stabilizing the interaction with 53BP1	Lin et al. (2015)
	PRMT5	PRMT5 promotes the methylation of TRIM28 at the R308 site	di Caprio et al. (2015)
	SMURF2	SMURF2 binds to TRIM28 and promoted its ubiquitination and degradation	Shah et al. (2022)
	RNF4	RNF4 stabilizes the abundance of TRIM28 in cells by promoting the ubiquitination degradation of SUMOylated TRIM28	Kuo et al. (2014)
	SENP7	SEN7 promotes the deSUMOylation of TRIM28, thereby promoting chromosome relaxation under DDR.	Garvin et al. (2013)
	LMP1	LMP1 binds to TRIM28 and promoted its SUMOylation	Bentz et al. (2015)

The PTMs of TRIM28 are particularly important for their molecular functions, especially phosphorylation. Currently, research has found that TRIM28 is phosphorylated at multiple serine and threonine sites, such as S440, S473, S501, S824, Y44, Y458, and Y517, inhibiting its gene transcriptional inhibitory activity (Czerwińska et al., 2017a). Phosphorylation of S473 and S824 (pS473 and pS824) is particularly important. DNA damage activates Ataxia-Telangiectasia Mutated (ATM) kinase, which promotes DDR by phosphorylating several specific substrates to trigger damage response pathways (Li and Cucinotta, 2020). ATM promotes the phosphorylation of TRIM28 at S824 and S473, disrupting the interaction between TRIM28 and chromatin remodeling factors, thereby promoting DDR (Ziv et al., 2006; Goodarzi et al., 2011). Multiple studies have shown that pS824-TRIM28 causes chromosome relaxation, thereby promoting DDR and downstream gene transcription (Ziv et al., 2006). Interestingly, pS824-TRIM28 promotes cancer cell growth by regulating chromatin relaxation (Bhatia et al., 2013). In melanoma cancer cells, the MAGE protein upregulates ATM-dependent pS824-TRIM28 by promoting the binding of TRIM28 to ATM, thereby enhancing DDR and promoting tumor progression (Bhatia et al., 2013). pS473-TRIM28 is also involved in effective DDR and cell survival (Chang et al., 2008; Blasius et al., 2011; Magni et al., 2015). Depending on the type of DNA damage that occurs, TRIM28 is mediated by different phosphorylation kinases at the S473 site. In etoposide- or IR-induced stress responses, Chk2 (Checkpoint kinase 2) is the main kinase responsible for TRIM28 phosphorylation at the S473 site, while Chk1 (Checkpoint kinase 1) is an essential kinase responsible for TRIM28 phosphorylation at the S473 site induced by UV radiation (Blasius et al., 2011; Magni et al., 2015). Moreover, pS473-TRIM28 induced by DNA damage also promotes the interaction between TRIM28 and E2F1 (E2 promoter binding factor 1), thereby inhibiting the ability of E2F1 to activate apoptosis (Hu et al., 2012). Additionally, many other kinases have been shown to phosphorylate TRIM28. These include serine/threonine kinases p38MAPK, RIPK3 (receptor interacting serine/threonine kinase 3), NEK9 (NIMA related kinase 9), mTORC1 (mechanistic target of rapamycin complex 1) and DNA-dependent protein kinase catalytic subunits) (Shen et al., 2017; Agarwal et al., 2021; Park et al., 2021; Yang et al., 2022; Lu et al., 2023). Furthermore, PP2A dephosphorylates TRIM28 at S824 in prostate cancer (PCa) cells (Mita et al., 2016).

Moreover, the transcriptional inhibition activity of TRIM28 also depends on SUMOylation of at least three lysine residues (K554, K779, and K804). Interestingly, PHD and bromodomain serve as SUMO E3 ligases within the TRIM28 molecule, where PHD binds to UBC9 (SUMO E2 enzyme) and synergistically promotes the SUMOylation of TRIM28 (Ivanov et al., 2007). LMP1, the main viral oncoprotein of the Epstein–Barr virus (EBV), promotes SUMOylation of TRIM28 by binding to TRIM28 protein, thereby inhibiting EBV gene transcription and helping to maintain stable EBV latency (Bentz et al., 2015). Garvin et al. found that SENP7 (SUMO protease 7) promotes the deSUMOylation of TRIM28, thereby promoting chromosome relaxation under DDR (Garvin et al., 2013).

In addition, Lin et al. discovered four potential acetylation sites of TRIM28 through mass spectrometry analysis: K266, K377, K469, and K770 (Lin et al., 2015). During DDR, SIRT1 (Sirtuin 1) promotes the deacetylation of TRIM28, thereby stabilizing its interaction with 53BP1, leading to an increase in the formation of 53BP1 focal points responsive to DNA damage and promoting non-homologous end joining (NHEJ)-mediated DNA repair (Lin et al., 2015). Furthermore, PRMT5 (Protein arginine methyltransferase 5) promotes the methylation modification of TRIM28 at the R308 site, thereby preventing the interaction between TRIM28 and the KRAB-ZFP protein, ZNF224 (di Caprio et al., 2015).

SMURF2 (SMAD-specific E3 ubiquitin ligase 2) directly binds to TRIM28 to promote its ubiquitination (Shah et al., 2022). Interestingly, in normal cells, SMURF2 has a negative impact on TRIM28 expression, whereas SMURF2 stabilizes TRIM28 in tumor cells (Shah et al., 2022). Current research indicates that the relationship between ubiquitination and SUMOylation is highly complex and interactive (Li et al., 2023). RNF4 (Ring finger protein 4), a SUMO-targeted ubiquitin ligase, stabilizes the abundance of TRIM28 in cells by promoting the ubiquitination degradation of SUMOylated TRIM28 (Kuo et al., 2014).

## 3 Roles of TRIM28 in cancers

**FIGURE 2:**
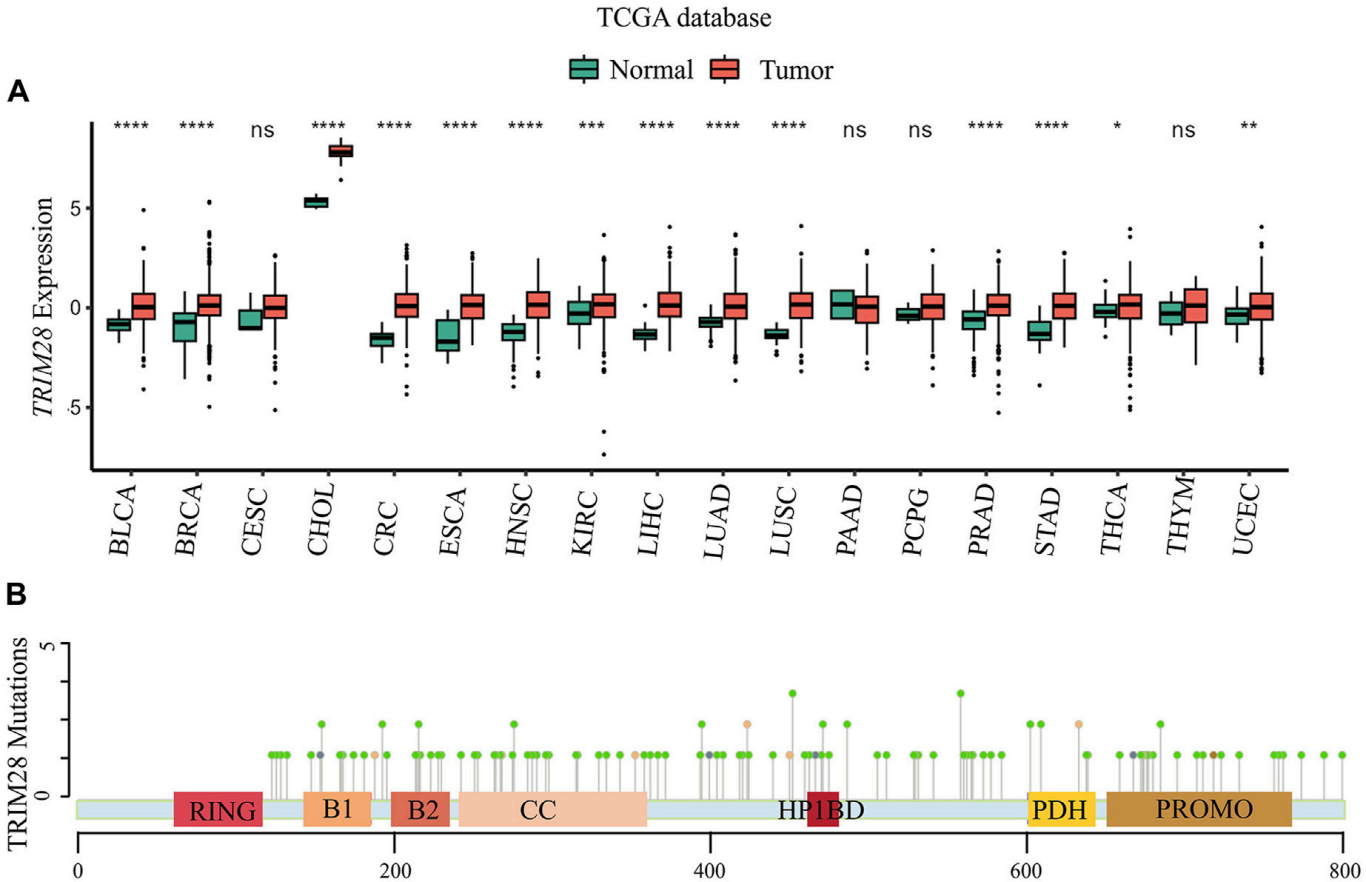
The expression and mutation of the *TRIM28* in human cancer **(A)** The expression of the *TRIM28* in human cancer: The TRIM28 gene expression dataset from The Cancer Genome Atlas (TCGA) database was extracted to explore its differential expression between tumors and adjacent normal tissues (data from the TIMER2.0 database http://timer.cistrome. org). Boxplots represent the distribution of gene expression levels and the statistical significance of differential expression was assessed using the Wilcoxon test. An asterisk indicates a p-value less than 0.05, two asterisks indicate a *p*-value less than 0.01, and three asterisks indicate a *p*-value less than 0.001. As illustrated in the figure, TRIM28 was significantly overexpressed in BLCA, BRCA, CHOL, CRC, ESCA, HNSC, KIRC, LIHC, LUAD, LUSC, PRAD, STAD, THCA, and UCEC. THCA, thyroid carcinoma; LUSC: Lung squamous cell carcinoma; BLCA: Bladder urothelial carcinoma; LIHC: Liver hepatocellular carcinoma; PRAD: Prostate adenocarcinoma; KIRC: Kidney renal clear cell carcinoma; UCEC: Uterine corpus endometrial carcinoma; LUAD: Lung adenocarcinoma; STAD: Stomach adenocarcinoma; BRCA: Breast invasive carcinoma; CESC: Cervical squamous cell carcinoma and endocervical adenocarcinoma; CHOL: Cholangiocarcinoma; ESCA: Esophageal carcinoma; HNSC: Head and Neck squamous cell carcinoma; PAAD: Pancreatic adenocarcinoma; PCPG: Pheochromocytoma and Paraganglioma; THYM: Thymoma; CRC: Colorectal Cancer. **(B)** The point mutation of TRIM28 in cancer: Point mutation data of TRIM28 protein displayed on the cBioPortal platform (https://www.cbioportal.org/) in all cancers.

To investigate the role of TRIM28 in cancer, we utilized a web platform (http://solvinglab.com.cn/) to study TCGA (The Cancer Genome Atlas) data and found that *TRIM28* mRNA levels were highly expressed in multiple cancers (Figure 2A). We found significant mutations in *TRIM28* in multiple tumor tissues, especially in endometrial cancer (EC), with a mutation probability of 6.25% (Table 2). Moreover, we used the cBioPortal platform (https://www.cbioportal.org/) to statistically analyze all point mutation of TRIM28 in cancer (Table 2) (Figure 2B). In addition, TRIM28 has different mutations in various types of cancers, mainly missense mutations. Therefore, we believe that it is necessary to study cancer-related mutations of TRIM28, which can further clarify its clinical significance.

**TABLE 2:** The mutations of TRIM28.

Cancer type	Mutation frequency in cancer, %	Protein change	Mutation type
LC	1.02	E583V; P585A; R629H; V808L; K238N	Missense mutation
BCa	1.41	E583K; M378I; M303I; A349T; Q288*	Missense mutation
HC	1.36	R374Q; D688Y; S279N; Q182R; D225V	Missense mutation
PCa	0.81	S667L; E491D	Missense mutation
RCC	0.53	M443I; T154S	Missense mutation
EC	6.25	E131D; E715K; R767H; D128Y; V383M; E388K; L300Q; D225N; S697*; R412H; M297V; V707A; R702W; S459T; V553A; P534S; S823I; E583D; C666Y; L173M; D636N; K275T; N358D; E411D; R262C; G480S; R483H; S598G; D189N; R591H; R472H	Missense mutation
		X280 splice; X196 splice; X661 splice; X703 splice	Splice Site
LC	1.07	V508L; V287I; R201L; P590S; I552V; M796I	Missense mutation
		X368_splice	Splice Site
GC	2.53	E555Q; R629H; Y755C; H344Y; Q288R; A528D; C224R; I743T	Missense mutation
		R492Qfs*9	Frame Shift Ins
		X442_splice	Splice Region
BC	0.93	E175K; S437C; R412C; S223G; R311H; S417*; S439R; Q233H; E715K; R706C	Missense mutation
		P161Qfs*18	Frame Shift Del
CRC	1.17	C704Y; R487*; G610C	Missense mutation
CC	2.09	F134L; E793K; D496N; R330Q	Missense mutation

**FIGURE 3:**
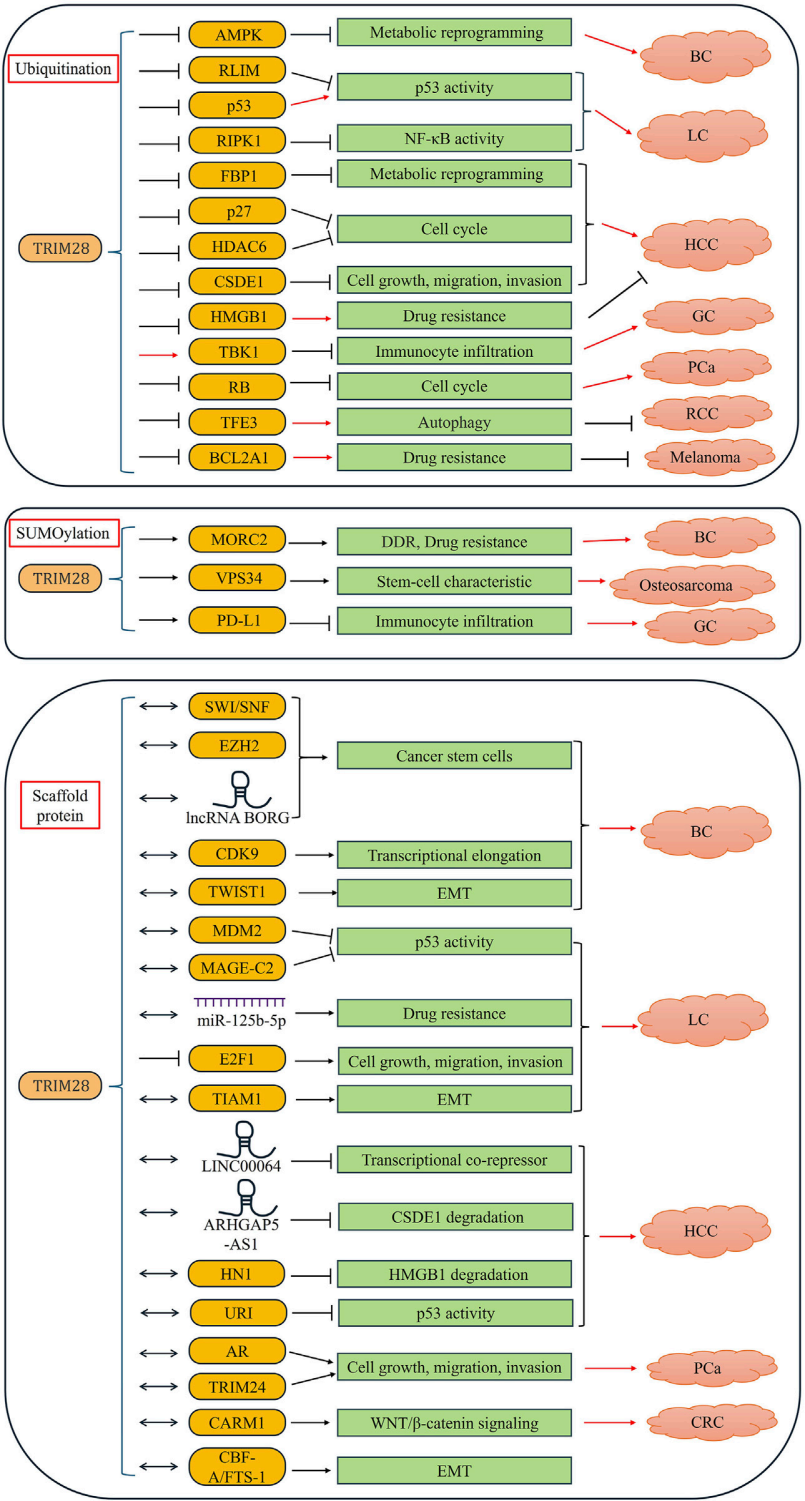
The role of TRIM28 in cancer TRIM28 protein, a ubiquitin E3 ligase, SUMO E3 ligase, or skeletal protein, plays an important role in the occurrence and development of tumors (including breast cancer, lung cancer, stomach cancer, liver cancer, colorectal cancer, prostate cancer, renal clear cell carcinoma, osteosarcoma melanoma, and neuroblastoma).

In addition, TRIM28 protein, a ubiquitin E3 ligase, SUMO E3 ligase, or skeletal protein also plays a key role in the development of tumors and tumorigenesis (Table 3) (Figure 3). The following are examples of different subtypes of cancer in which TRIM28 is considered to play a key role.

**TABLE 3:** The cancer-associated TRIM28 interacting proteins.

Role	Interacting protein	Biological functions	Oncogenic or tumor suppressive	Reference
Ubiquitin E3 ligase	AMPK	TRIM2 promotes ubiquitination degradation of AMPK	Oncogenic	Pineda and Potts (2015)
	RLIM	TRIM28 promotes the ubiquitination degradation of RLIM, thereby stabilizing the level of MDM2	Oncogenic	Jin et al. (2021)
	p53	TRIM28 independently ubiquitinates p53 as an E3 ligase activated by MAGE-C2	Oncogenic	Doyle et al. (2010)
	RIPK1	TRIM28 directly binds to RIPK1 and promotes the K63-linked ubiquitination of RIPK1	Oncogenic	Liang et al. (2023)
	FBP1	TRIM28 promotes ubiquitination degradation of FBP1	Oncogenic	Jin et al. (2017)
	p27	TRIM28 promotes ubiquitination of p27 to promote its degradation	Oncogenic	Zhang et al. (2021b)
	HDAC6	RIM28 can promote the ubiquitination degradation of HDAC6	Oncogenic	Li et al. (2021)
	CSDE1	TRIM28 catalyzes the polyubiquitination and proteasome degradation of CSDE1	Oncogenic	Liu et al. (2022b)
	HMGB1	TRIM28 binds to HMGB1 and promotes its ubiquitination mediated lysosomal degradation	Oncogenic	Wang et al. (2022)
	TBK1	TRIM28 promotes K63 polyubiquitination modification of TBK1	Oncogenic	Ma et al. (2023)
	RB	TRIM28 binds and promotes the polyubiquitination and degradation of phosphorylated RB by CDK4/6	Oncogenic	Huang et al. (2023)
	TFE3	TRIM28 promotes the ubiquitination and degradation of TFE3, and inhibits RCC cell proliferation	Tumor suppressive	Song et al. (2023)
	BCL2A1	TRIM28 binds to BCL2A1 on mitochondria and promotes its ubiquitination degradation	Tumor suppressive	Lionnard et al. (2019)
SUMO E3 ligase	MORC2	TRIM28 promotes SUMOylation of MORC2 at the K827 site	Oncogenic	Zhang et al. (2023a)
	VPS34 (PI3KC3)	TRIM28 promotes SUMOylation modification of VPS34 by forming a complex with PVT-1	Oncogenic	Tsang et al. (2022)
	PD-L1	TRIM28 promotes the SUMOylation of PD-L1, thereby inhibiting its ubiquitination degradation	Oncogenic	Ma et al. (2023)
Scaffold protein	EZH2 and SWI/SNF	TRIM28 interacts with EZH2 and SWI/SNF to promoting promotes BCSC enrichment and maintenance	Oncogenic	Li et al. (2017)
	CDK9	TRIM28 stimulates effective transcriptional elongation by recruiting CDK9	Oncogenic	Yang et al. (2022)
	TWIST1	TRIM28 can stabilize TWIST1 by combining with it	Oncogenic	Wei et al. (2016)
	METTL3	TRIM28 promotes m6A modification of MYCN mRNA by binding to METTL3, thereby upregulating the stability of MYCN mRNA	Oncogenic	Yang et al. (2024)
	CARM1	TRIM28 interacts with CARM1 and protects CARM1 from proteasome mediated degradation	Tumor suppressive	Cui et al. (2019)
	MDM2	TRIM28 can bind to MDM2, synergistically promoting p53 ubiquitination and degradation	Oncogenic	Wang et al. (2005)
	TIAM1	TRIM28 can form a complex with TIAM1, further promoting EMT	Oncogenic	Ginn et al. (2023)
	AR	TRIM28 promotes the transcriptional activity of AR	Oncogenic	Fong et al. (2018)
	TRIM24	TRIM28 also prevents degradation of TRIM24 by SPOP	Oncogenic	Bykov et al. (2018)
	E2F1	TRIM28 binds to E2F1, promoting E2F1 inactivation and inhibiting cell apoptosis	Oncogenic	Zhang et al. (2023b)
	MAGE-C2	TRIM28 interaction with MAGE-C2 increases co-precipitation of TRIM28 with ATM upon genotoxic stress	Oncogenic	Bhatia et al. (2013)
	URI	URI promotes the ubiquitination degradation of p53 by binding to TRIM28	Oncogenic	Mita et al. (2016)
	CBF-A/FTS-1	TRIM28 combines with CBF-A/FTS-1 to promote EMT	Oncogenic	Venkov et al. (2007)

### 3.1 The carcinogenic role of TRIM28

#### 3.1.1 TRIM28 in BC

BC is the most common malignancy in women, which seriously damages their health (Sung et al., 2021). Although various antitumor treatments have, to some extent, slowed down the progression of BC, certain subtypes of BC are not able to achieve good therapeutic effects due to the high heterogeneity of pathological features (Sung et al., 2021). The expression of TRIM28 at mRNA and protein level have been found to be significantly higher in BC tissues (Hao et al., 2017; Zhang W. et al., 2021). Furthermore, immunohistochemistry (IHC) staining confirmed higher expression of TRIM28 and pS824-TRIM28 in highly invasive cancer subtypes (HER2, Basal-like) (Addison et al., 2015). In addition, TRIM28 depletion reduced BC formation in mouse tumor models (Czerwińska et al., 2017b).

The tumor suppressor AMPK is a master sensor and regulator of the cellular energy status (Chaube and Bhat, 2016). Pineda *et al.* demonstrated that TRIM28 forms a cancer-specific ubiquitinase with MAGE protein, which ubiquitinates and degrades AMPK (Pineda and Potts, 2015). CzerwinSka *et al.* found a significant increase in AMPK protein levels in BC allografts with TRIM28 deletion (Czerwińska et al., 2017b).

DNA damage is caused by a variety of factors, including chemical carcinogens, radiation, and the subsequent initiation of DDR (Jackson and Bartek, 2009). MORC2 (Microrchidia CW-type zinc finger 2) plays a central role in DDR (Li et al., 2012). Notably, under the action of the DNA-damaging agent Dox (doxorubicin), TRIM28 promotes SUMOylation of MORC2 at the K827 site, thereby recruiting CSK21 (casein kinase II subunit alpha) and inducing the activation of DNA-PKcs, promoting DDR (Zhang FL. et al., 2023). At the same time, Zhang *et al.* used a xenograft transistor model to discover that the SUMOylation-deficient mutant MORC2 enhances the sensitivity of BC cells to DNA-damaging chemotherapeutic drugs (Zhang FL. et al., 2023). Interestingly, Lee *et al.* found that DOX was able to inhibit the SUMOylation of TRIM28 itself, thereby promoting the transcription of p21 and, thus, the growth of the BC cell line MCF-7 (Lee et al., 2007).

Cancer stem cells (CSCs) are present in various types of cancer. Studies have shown that tumor growth is driven by a small number of CSC (Batlle and Clevers, 2017). TRIM28 plays an important role in BC stem cells (BCSCs) (Czerwińska et al., 2017b). Downregulation of TRIM28 expression reduces BCSCs’ ability of BCSCs to self-renew and inhibits tumor growth (Czerwińska et al., 2017b). In MCF-7 cells, TRIM28 interacts with EZH2 (Enhancer of Zeste Homolog 2) and SWI/SNF (ATP-dependent chromatin remodeling complex, also called BAF) to promote BCSC enrichment and maintenance (Li et al., 2017). TRIM28 binds to lncRNA BORG (long non-coding RNA BMP/OP-Responsive Gene) to form the lncRNA BORG/TRIM28 complex, which induces self-renewal and expansion of BCSCs (Parker et al., 2021). Hypoxia is a prominent feature of the tumor microenvironment and a hallmark of many solid tumors (Harris, 2002). HIF-1α binds to the hypoxia-responsive elements (HREs) of the target gene, recruiting Pol II (RNA polymerase II) to stimulate transcription of the target gene under hypoxia (Galbraith et al., 2013; Semenza, 2014). Although Pol II initiates transcription, it pauses approximately 30–60 nucleotides downstream of the transcription start site (Bunch et al., 2014). In BC cells, DNA-PKcs are recruited into the HRE by HIF-1α and promote the phosphorylation of TRIM28 at the S824 site. Phosphorylated TRIM28 recruits CDK9 (Yang et al., 2022). CDK9 deactivates Pol II by phosphorylation, thereby stimulating efficient transcriptional elongation (Yang et al., 2022). Deregulation of cellular energy, a characteristic feature of cancer, is primarily caused by mitochondrial dysfunction (Gomes et al., 2011). TRIM28 is phosphorylated by ROS-p38MAPK at the S473 site in BC cells that have been starved for a long time (Cheng et al., 2016). Moreover, pS473-TRIM28 downregulates MFN2 (Mitofusin-2), thus reducing the excessive fusion of mitochondria, enabling BC cells to survive in the changes in tumor microenvironment (TME), thus promoting tumor growth (Cheng et al., 2016). TWIST1 (Twist related protein 1) is a tumor protein that plays an important role in tumor metastasis and drug resistance (Yang et al., 2004). Mechanistically, TWIST1 enhances metastasis by promoting epithelial-to-mesenchymal transition (EMT) (Kang and Massagué, 2004). TRIM28 can stabilize TWIST1 by combining with it and promoting BC metastasis (Wei et al., 2016). Moreover, TRIM28 upregulates intracellular TWIST1 protein levels, but does not affect TWIST1 mRNA levels (Wei et al., 2016). In conclusion, these findings support a key role for TRIM28 in regulating BC tumorigenesis and progression.

#### 3.1.2 TRIM28 in GC

GC is one of the deadliest malignant tumors, with a 5-year survival rate of approximately 20% (Sung et al., 2021). Upregulation of TRIM28 protein and mRNA levels has been detected in GC tissues compared to normal tissues (Yokoe et al., 2010). Simultaneous survival analysis showed that patients with high TRIM28 expression had poorer prognosis (Yokoe et al., 2010).

EBV is a pathogenic virus found in many malignant tumors (Farrell, 2019). EBV has been detected in 10% of GC tissues (Burke et al., 1990). SNHG8, a member of the small nucleolar RNA host gene (SNHG) family, is an oncogene in GC (Zhang et al., 2020). SNHG8 is significantly overexpressed in EBV-associated GC (EBVaGC) tissue and promotes the proliferation and migration of EBVaGC cells (Zou et al., 2021). Further research has found that TRIM28 is crucial for SNHG8 mediated malignant behavior (Zou et al., 2021). Mechanistically, SNHG8 sponges have miR-512-5p and upregulate TRIM28, thereby exerting a cancer-promoting effect (Zou et al., 2021). Cancer associated fibroblasts (CAFs) directly or indirectly affect cancer progression and tumor immunity by secreting various proteins (Chen et al., 2021). SLIT2 (Slit guidance ligand 2) is an axon-guiding protein secreted by CAFs, which promotes the kinase activity of NEK9 in GC cells by binding to the surface receptor ROBO1 (Roundabout guidance receptor 1) of GC cells (Lu et al., 2023). As mentioned earlier, TRIM28 is phosphorylated by NEK9 (Lu et al., 2023). Phosphorylation downregulates the transcriptional inhibition function of TRIM28 and promotes gene transcription, thus enabling GC cells to obtain excessive migration ability (Lu et al., 2023).

Targeted immune checkpoints have recently been used in GC therapy (Xie et al., 2021). Recent studies have shown that the response to PD-1/PD-L1 treatment may be related to the expression level of PD-L1 in tumor cells (Zhang et al., 2018; Shen et al., 2022). TRIM28 is an important regulatory factor for PD-L1 expression in GC cells (Ma et al., 2023). Mechanistically, TRIM28 inhibits the ubiquitination and degradation of PD-L1 by promoting PD-L1 SUMOylation, thereby stabilizing PD-L1 within cells. In addition, TRIM28 promotes non-degradable polyubiquitination of TBK1 (TANK-binding kinase 1), activating the TBK1-mTOR pathway, thereby enhancing PD-L1 transcription (Ma et al., 2023). Simultaneously, ectopic TRIM28 expression promotes tumor growth in mice and inhibits T-cell activation (Ma et al., 2023). Interestingly, TRIM28 promotes drug resistance in GC cells by influencing Wnt/β-catenin signaling, indicating that TRIM28 is a promising drug target and potential prognostic factor (Ning et al., 2023).

#### 3.1.3 TRIM28 in PCa

PCa is the second most common cancer in men and the eighth leading cause of cancer death (Rawla, 2019). Androgen deprivation therapy (ADT) has shown promising results in the treatment of PCa (Dai et al., 2017). However, most patients eventually relapse because of castration-resistant PCa (CRPC) (Dai et al., 2017). *TRIM28* is significantly upregulated in CRPC, and TRIM28 knockdown inhibits the growth of PCa xenografts (Fong et al., 2018). Further mechanistic studies have shown that TRIM28 enhances AR signaling in PCa cells and that TRIM28 inhibits the degradation of TRIM24 by SPOP, further promoting the transcriptional activity of AR (Van Tilborgh et al., 2013; Fong et al., 2018). Yende *et al.* showed that TRIM28 promotes the expression of proximal luminal lineage cell markers in PCa cells (Yende et al., 2023). RB (Retinoblastoma) is an important tumor suppressor protein that inhibits cell cycle (Engeland, 2022). RB phosphorylation plays a crucial role in mediating the inhibitory effect of RB (Engeland, 2022). Huang *et al.* found that TRIM28 can bind and promote polyubiquitination and degradation of phosphorylated RB by CDK4/6, thereby promoting the progression of PCa (Huang et al., 2023). Overall, these studies indicate that TRIM28 may be a potential tumor promoter in PCa.

#### 3.1.4 TRIM28 in CC (cervical cancer) and OC (ovarian cancer)

At present, the incidence of CC and OC is still rising worldwide, posing a serious threat to women’s health (Lajer et al., 2010). However, research on TRIM28 expression in CC and OC remains limited. Studies have shown that TRIM28 is highly expressed in CC and OC tissues (Cui et al., 2014; Li et al., 2018). TRIM28 promotes CC cell proliferation by activating the mTOR signaling pathway (Li et al., 2018). In OC, TRIM28 recruits E2F1 to activate the PI3K/AKT/mTOR signaling pathway and promote OC development (Zhang F. et al., 2023). Deng *et al.* found that knocking down TRIM28 downregulated the Wnt/β-catenin signaling pathway, thereby inhibiting the migration, invasion, and EMT of OC cells (Deng et al., 2017). Moreover, TRIM28 inhibits the antitumor immune microenvironment by binding to SETDB1 (Lin et al., 2021). However, these studies have not focused on the ubiquitination E3 function of TRIM28 and have not fully revealed the important role of TRIM28 in CC and OC. Further research should be conducted to elucidate the carcinogenic role of TRIM28.

#### 3.1.5 TRIM28 in other cancers

Pancreatic cancer (PC) is one of the deadliest malignant tumors (Cai et al., 2021). Yu *et al.* found that TRIM28 is highly expressed in PC tissues and is associated with poor prognosis (Yu et al., 2014). The overexpression of TRIM28 promotes EMT and increases the migration and invasion of PC cells *in vitro* and *in vivo* (Yu et al., 2014). Neuroblastoma (NB) is the most common extracranial solid tumor in childhood (Qiu and Matthay, 2022). *MYCN* amplification is characteristic of high-risk NB (Otte et al., 2020). TRIM28 is highly expressed in NB (Yang et al., 2024). Moreover, TRIM28 promotes m^6^A modification of MYCN mRNA by binding to METTL3 (Methyltransferase-like 3), thereby upregulating the stability of MYCN mRNA (Yang et al., 2024). Glioma is the most common intracranial tumor and is characterized by high malignancy and poor prognosis (Ostrom et al., 2015). Molecular studies have found that *TRIM28* is significantly elevated in glioma samples compared to normal brain tissue and is positively correlated with tumor malignancy (Qi ZX. et al., 2016; Su et al., 2018). High TRIM28 expression may indicate poor prognosis in patients with glioma (Qi ZX. et al., 2016). TRIM28 knockout inhibits autophagy in glioblastoma cells and increases p21 expression, inducing tumor cell cycle arrest (Peng et al., 2019). TRIM28 has also been reported to function in B-cell non-Hodgkin lymphoma (B-NHL). B-NHL is progressive lymphadenopathy (Moleti et al., 2020). Zhang *et.al.* found that high TRIM28 expression was positively associated with poor survival in patients with B-NHL (Mohan et al., 2016). In addition, TRIM28 promotes the proliferation of B-NHL cells by modulating cell cycle progression (Mohan et al., 2016). Osteosarcoma (OS) is a primary bone malignancy (Liu et al., 2021). Vps34, an E3 ubiquitination ligase, can promote ubiquitination degradation of TSC2 (Tuberous sclerosis complex 2), thereby promoting carcinogenic transformation (Mohan et al., 2016). In osteosarcoma, TRIM28 promotes the SUMOylation modification of VPS34 by forming a complex with PVT-1 (Plasmacytoma variant translocation-1), further enhancing the ubiquitination and degradation of TSC2, thereby enhancing the self-renewal and stem cell phenotype of osteosarcoma cells (Tsang et al., 2022).

### 3.2 The anticancer effect of TRIM28

#### 3.2.1 TRIM28 in KC (Kidney cancer)

In contrast to the oncogenic role of TRIM28 protein in many human cancers described above, the tumor-suppressive function of TRIM28 has been confirmed in KC. Research has shown that overexpression of TRIM28 inhibits the growth of renal cell carcinoma (RCC) cells, and high TRIM28 protein and mRNA expression is associated with better OS in RCC patients (Song et al., 2023). Biochemically, TRIM28 promotes ubiquitination and degradation of TFE3 and inhibits RCC cell proliferation (Song et al., 2023). Moreover, *TRIM28* has been identified as a susceptibility gene for Wilms’ tumor (WT), with germline pathogenic variants identified in approximately 1% of isolated and 8% of familial WT cases (Hol et al., 2021). Moreover, Wegert *et al.* used immunohistochemical techniques and found that 44.4% (56/126) of WT cases exhibited TRIM28 deficiency (Wegert et al., 2024). This indicates that the precise detection of TRIM28 may contribute to the early detection of WT (Hol et al., 2021; Wegert et al., 2024). Taken together, these findings suggest that TRIM28 has an antitumor effect in kC cells.

### 3.3 The role of TRIM28 remains to be determined

#### 3.3.1 TRIM28 in lung cancer (LC)

LC is one of the leading causes of cancer-related deaths worldwide (Sung et al., 2021). TRIM28 protein and mRNA are highly expressed in LC tissues (Liu et al., 2018). Although Chen *et al.* found that high TRIM28 expression was associated with increased overall survival in early LC and reduced cell proliferation in model LC cell lines (Chen et al., 2012), another study showed that TRIM28 knockdown inhibited LC cell proliferation, promoted cell apoptosis, and inhibited the growth of subcutaneous LC grafts in mice (Liu et al., 2018).

P53 is the most important tumor suppressor factor involved in various cellular signaling processes (Bykov et al., 2018). In human cells, MDM2 (mouse double minute 2) is the main regulatory factor of p53 (Haupt et al., 1997). MDM2 regulates the abundance of p53 by directly binding to p53 and promoting its ubiquitination and degradation (Haupt et al., 1997). Wang *et al.* found that TRIM28 can bind to MDM2 and synergistically promote p53 ubiquitination and degradation (Wang et al., 2005). Meanwhile, TRIM28 as a MAGE, activates ubiquitin E3 ligase and independently ubiquitinates p53 (Doyle et al., 2010). In addition, Liu *et al.* found that MAGE also inhibits the ubiquitination degradation of p53 by directly binding to MDM2 (Liu Y. et al., 2022). TRIM28 overexpression competitively binds to MAGE, thereby promoting p53 ubiquitination, degradation, and cell proliferation (Liu Y. et al., 2022). Moreover, *in vitro* experiments have shown that TRIM28 can also stabilize the protein level of MDM2 by promoting the ubiquitination degradation of RLIM, a ubiquitin E3 ligase of MDM2, thereby further downregulating the low expression level of p53 and ultimately promoting the proliferation, migration, and invasion of LC cells (Jin et al., 2021). Avoiding immune responses and stimulating inflammation caused by tumors are among several strategies adopted by cancer cells to maintain proliferation and progression (Whiteside, 2008; Schreiber et al., 2011). Tumor cells escape the immune system by promoting the activation of tumor suppressive cells, such as regulatory cells (Tregs) and myeloid-derived suppressor cells (MDSCs) (Whiteside, 2008; Schreiber et al., 2011; Zhao et al., 2021). Bioinformatic studies have shown a negative correlation between TRIM28 and immune infiltration (Liu et al., 2020). Liang *et al.* found that the expression of TRIM28 is positively correlated with MDSC infiltration in LC (Liang et al., 2023). In addition, silencing TRIM28 can enhance the efficacy of anti-PD-1 immunotherapy by reshaping the inflammatory TME (Liang et al., 2023). Mechanistically, TRIM28 directly binds to RIPK1 (Receptor-interacting protein kinase 1) and promotes K63-linked ubiquitination of RIPK1, thereby activating the NF-κB (nuclear factor NF-Kappa-B) pathway (Liang et al., 2023). Further experiments revealed that TRIM28 activates the NF-κB pathway and upregulates CXCL1 (C-X-C motif ligand 1) expression (Liang et al., 2023). CXCL1 can bind to CXCR2 (C-X-C chemokine receptor 1) on MDSCs, promoting their migration into the TME (Liang et al., 2023). TRIM28 knockout increased the responsiveness of anti-PD-1 therapy in immunocompetent mice (Liang et al., 2023).

Cisplatin (DDP) is a commonly used chemotherapeutic drug for NSCLC, which promotes cell death by inducing DNA damage in tumor cells (Kryczka et al., 2021). However, long-term DDP treatment can increase the drug resistance of LC cells, leading to tumor progression (Kryczka et al., 2021). Tan *et al.* found that miR-125b-5p induced resistance to DDP in LC cells (Tan et al., 2022). Further research has found that TRIM28 can promote miR-125b-5p and plays an important role in DDP resistance in LC patients (Tan et al., 2022). In addition, TRIM28 knockdown induces cell apoptosis by increasing E2F1 inactivation and downregulating the sensitivity of LC cells to etoposide therapy (Liu et al., 2017). Similar to BC, TRIM28 is also associated with EMT activation. In NSCLC cells, TRIM28 promotes TGF-β-induced EMT by silencing E-cadherin expression, thereby promoting tumor cell migration and invasion (Chen et al., 2014). Moreover, TRIM28 can form a complex with TIAM1 (T-cell invasion and metastasis-inducing protein 1) to silence E-cadherin expression and promote EMT in LC cells (Ginn et al., 2023). In summary, these findings support the crucial role of TRIM28 in the different stages of LC development, and TRIM28 may be a potential clinical therapeutic target.

#### 3.3.2 TRIM28 in HCC (hepatocellular carcinoma)

HCC is one of the most common cancers (Sung et al., 2021). TRIM28 plays a dual role in HCC, being associated with distant metastasis in HCC patients and is closely related to poor prognosis in HCC patients, while in a mouse HCC model, it acts as a tumor suppressor to prevent tumorigenic transformation (Bojkowska et al., 2012; Wang et al., 2016).

FBP1 (Fructose-1,6-diphosphatase) has recently been identified as a tumor suppressor in HCC (Yang et al., 2017). TRIM28 induces metabolic reprogramming in HCC cells by binding to FBP1 and promoting its ubiquitination degradation, thereby promoting the occurrence and development of HCC (Jin et al., 2017). HDAC6 (Histone deacetylase 6) is a transcriptional regulatory factor, playing a crucial role in transcriptional regulation and cell cycle progression (Pulya et al., 2021). HDAC6 inhibits the progression of HCC by forming a transcriptional inhibition complex with TRIM28 (Oo et al., 2020; Li et al., 2021). Moreover, TRIM28 maintains the intracellular abundance of HDAC6 by promoting the ubiquitination degradation of HDAC6 (Li et al., 2021). LINC00064, an antisense lncRNA, enhances the interaction between HDAC6 and TRIM28, thereby accelerating the degradation of HDAC6 (Li et al., 2021). In contrast, LINC00064 binds to the RBCC domain of TRIM28, inhibiting the formation of the HDAC6-TRIM28 transcriptional co-repressor protein complex and promoting HCC progression (Li et al., 2021). CSDE1 (Cold shock domain-containing protein E1) is an RNA-binding protein that plays a vital role in tumorigenesis by coordinating oncogenic RNA regulators such as *VIM* and *RAC1* genes (Wurth et al., 2016; Fabbri et al., 2021). Notably, TRIM28 catalyzes the poly-ubiquitination and degradation of CSDE1 (Liu J. et al., 2022). Interestingly, ARHGAP5‐AS1, an lncRNA, attenuates the interaction between CSDE1 and TRIM28, which prevents the degradation of CSDE1, contributing to the progression of HCC (Liu J. et al., 2022).

Similar to LC, TRIM28 can also promote HCC progression by affecting the abundance of p53 protein in cells. Moreover, in HCC cells, URI activity and MUFA (Monounsaturated fatty acid) accumulation, and subsequently promoting cancer cell resistance to TKIs (Tyrosine kinase inhibitors) (Ding et al., 2023). Lenvatinib, a TKI, is the first-line treatment for HCC (Kudo et al., 2018). Current research indicates that *TRIM28* is a target gene for WDR4, and high TRIM28 expression is significantly associated with cell-acquired stemness and lenvatinib resistance in HCC (Han et al., 2023). Bortezomib (BTZ) is a selective proteasome inhibitor that has shown promising results in the treatment of HCC (Saeki et al., 2013). The proteasome is composed of a 20S subunit and one or two 19S regulatory subunits (Saeki et al., 2013). Under the action of BTZ, TRIM28 significantly upregulates the expression of the 20s subunit (Zhang et al., 2022). Further research has shown that, under the action of BTZ, TRIM28 enters the nucleus and activates the expression of various proteasome subunits, thereby making HCC cells resistant to BTZ (Zhang et al., 2022). Moreover, nuclear TRIM28 supports the proliferation and migration of HCC cells, partially by promoting the ubiquitination degradation of p27 to promote cell cycle progression (Zhang RY. et al., 2021). Therefore, TRIM28 may be a viable target for novel anticancer agents aimed at HCC inhibition.

Platinum-based drugs have significant inhibitory effects on human HCC cells. Oxaliplatin is an important drug for the clinical chemotherapy of HCC (Li et al., 2022). Unlike in LC cells, where TRIM28 promotes cisplatin resistance, in HCC cells, TRIM28 promotes sensitivity to oxaliplatin treatment by binding to HMGB1 (high mobility group protein B1) and promoting ubiquitination-mediated lysosomal degradation (Wang et al., 2022). However, HN1 (Hematological and neurological expressed 1) can competitively bind to TRIM28, thereby inhibiting the degradation of HMGB1, promoting the proliferation and metastasis of HCC, and reducing chemotherapy sensitivity to oxaliplatin (Wang et al., 2022).

#### 3.3.3 TRIM28 in CRC (colorectal cancer)

CRC is the second leading cause of cancer-related death (Sung et al., 2021). *TRIM28* is upregulated in the interstitial tissue of patients with CRC, and its increased expression is associated with poor prognosis (Fitzgerald et al., 2013). SIK2 (Salt induced kinase 2) is an oncoprotein that is upregulated in CRC, and knocking down SIK2 can weaken the proliferation, invasion, and glycolysis of CRC cells (Ni et al., 2021). The overexpression of TRIM28 can reverse the effects of silencing SIK2 (Ni et al., 2021). Similar to BC, the phosphorylation of TRIM28 at the S473 site induced by ROS-p38MAPK effectively promotes DDR and helps CRC cells fight exogenous ROS (Shen et al., 2017). However, Cui *et.al* found that TRIM28 protein levels were downregulated in CRC tissues and were associated with good prognosis (Cui et al., 2019). Interestingly, TRIM28 protein was significantly overexpressed in CRC exosomes. Mechanistically, TRIM28 interacts with CARM1 (Co-activator-associated arginine methyltransferase1) and protects CARM1 from degradation, thereby inhibiting WNT/β-catenin signaling to inhibit the migration and invasion of CRC cells (Cui et al., 2019). These results indicate that TRIM28 may exert certain antitumor effects in CRC, which remains to be elucidated in the future.

#### 3.3.4 TRIM28 in melanoma

Melanoma is a skin cancer caused by malignant melanoma, and its incidence is increasing rapidly worldwide. High TRIM28 mRNA expression is associated with the stem cell-like phenotype of melanoma cells and poor prognosis in melanoma patients (Czerwinska et al., 2020). Further research has found that TRIM28 knockdown induces the transition of melanoma from invasiveness to tumor growth (Nyberg et al., 2023). Mechanistically, TRIM28 promotes the invasiveness of melanoma cells and inhibits tumor growth by negatively regulating the expression of Jun B proto-oncogene (JUNB) (Nyberg et al., 2023). TRIM28 often forms complexes with MAGE to exert its ubiquitination E3 function. However, TRIM28 can positively regulate the abundance of MAGE proteins in melanoma cells (Song et al., 2018). BCL2A1 (B-cell lymphoma 2-related protein A1) is an anti-apoptotic member of the BCL-2 family and is associated with the resistance of melanoma cells to BRAF-targeted therapy (Haq et al., 2013). Lionnard *et al.* found that TRIM28 binds to BCL2A1 on the mitochondria and promotes its ubiquitination degradation, thereby upregulating the sensitivity of melanoma cells to BRAF-targeted therapy (Lionnard et al., 2019). These studies suggest that TRIM28 plays an important role in melanoma and may be a new therapeutic target for melanoma.

## 4 TRIM28 as a therapeutic target

As discussed, TRIM28 can regulate various biological functions, including DDR (Ziv et al., 2006; Goodarzi et al., 2011), EMT (Venkov et al., 2007; Wei et al., 2016; Ginn et al., 2023), maintaining cellular stemness (Czerwińska et al., 2017b; Li et al., 2017; Han et al., 2023). Thus, TRIM28 is a potential target for cancer treatment. In addition, the expression level of TRIM28 affects the drug resistance of tumor cells, which seriously affects the anticancer effects of treatments, such as chemotherapy, targeted therapy, and immunotherapy.

As mentioned earlier, TRIM28 may inhibit the sensitivity of tumor cells to chemotherapy. Knockdown of TRIM28 expression in LC cells promotes sensitivity to 5-FU, etoposide, and cisplatin (Damineni et al., 2017). TRIM28 weakens the sensitivity of HCC cells to BTZ by enhancing proteasome expression (Zhang et al., 2022). Bladder cancer (BCa) has a 70% prevalence of telomerase reverse transcriptase (TERT) promoter mutation, which is associated with poor patient prognosis (Kinde et al., 2013). Phosphorylation of TRIM28 by mTORC1 activates TERT transcription of mutant promoter alleles and promotes BCa cell growth (Agarwal et al., 2021). Ridaforolimus, an mTORC1 inhibitor, suppresses TRIM28 phosphorylation, hTERT expression, and cell viability (Agarwal et al., 2021). However, TRIM28 can enhance the sensitivity of HCC cells to oxaliplatin therapy by promoting the ubiquitination degradation of HMGB1 (Wang et al., 2022).

Targeted therapy is becoming increasingly important for the treatment of tumors. High TRIM28 expression promotes resistance of HCC to TKI therapy (Han et al., 2023). However, in melanoma, TRIM28 upregulates the sensitivity of melanoma cells to targeted BRAF therapy by promoting the degradation of BCL2A1 (Lionnard et al., 2019). In TRIM28 deficient cells, actinomycin D promoted p53 signal activation, indicating that TRIM28 is a probable target for p53 signal activation (Okamoto et al., 2006). JQ1 is one of the most extensively studied BET protein-selective inhibitors (Wang et al., 2018). Overexpression of TRIM28 leads to increased degradation of FBP1, which in turn inhibits the degradation of c-Myc, leading to increased resistance of PC cells to JQ1 (Wang et al., 2018).

Immunotherapy is becoming increasingly important for tumor treatment. As mentioned earlier, TRIM28 knockdown can enhance the efficacy of immunotherapy (Liang et al., 2023; Ma et al., 2023). Verteporfin is a small-molecule inhibitor of PD-L1 expression (Liang et al., 2020). Liang *et al.* found that verteporfin might inhibit PD-L1 expression by interfering with the interaction of TRIM28-IRF1 (Liang et al., 2020). TRIM28 is also involved in the development and activation of T cells (Tanaka et al., 2018). Gehrmann *et al.* found that TRIM28 can promote the differentiation of immature T cells into Tregs (Gehrmann et al., 2019). TRIM28 can inhibit the antitumor immune microenvironment (Liu et al., 2020). These studies suggest that the targeted inhibition of TRIM28 may effectively enhance the efficacy of tumor immunotherapy.

Radiation therapy can cause DNA breakage in tumor cells, thereby killing them (Moding et al., 2013). Downregulating the abundance of TRIM28 in tumor cells can increase the efficacy of radiotherapy, and ATM inhibitor (ATMi) drugs promote the sensitivity of glioma cells to radiotherapy by inhibiting TRIM28 phosphorylation (Golding et al., 2012; Lee et al., 2020).

Nanomaterials have been used in clinical trials (Kijanka et al., 2015). NB237, an anti-TRIM28 nanoparticle, significantly inhibits the invasion and growth of glioblastoma cells (Porčnik et al., 2021). However, there is currently a lack of small-molecule inhibitors targeting TRIM28, which may be an important direction for future research.

## 5 Discussion

Hanahan and Weinberg described ten characteristics of cancer, which are strategies that cancer cells acquire to enable survival, growth, and metastasis (Hanahan and Weinberg, 2011). Numerous studies suggest that TRIM28 is involved in cancer signaling pathways, particularly in promoting cell proliferation, immune evasion, inflammation, invasion and migration, and evasion of apoptosis. TRIM28 is not only highly expressed in multiple cancer tissues, but also promotes the degradation of multiple tumor suppressor proteins, such as p53 and AMPK, TRIM28 can upregulate the expression of oncogenes, such as AR, which promotes the development of most tumors. However, in RCC and early LC, TRIM28 plays an anti-cancer role, indicate that TRIM28 plays a dual role in the occurrence and development of tumors and is related to the background of the tumor, which requires further research. Moreover, TRIM28 knockdown can enhance the efficacy of chemotherapy, targeted therapy, and immunotherapy in cancer treatment. Meanwhile, the PTMs of TRIM28, particularly phosphorylation and SUMOylation, are crucial for its transcriptional inhibition, but their impact on its ubiquitin E3 activity is currently unclear. In addition, TRIM28 acts as both a SUMO and ubiquitin E3 ligase, cascading two different PTMs, and plays important roles in protein homeostasis and signal transduction. However, the reason for TRIM28’s selective ubiquitination or SUMOylation of substrates is currently unclear. However, this requires further investigation.
